# Prospective study of postoperative whole breast radiotherapy for Japanese large-breasted women: a clinical and dosimetric comparisons between supine and prone positions and a dose measurement using a breast phantom

**DOI:** 10.1186/s12885-016-2794-z

**Published:** 2016-09-29

**Authors:** Kana Takahashi, Madoka Morota, Yoshikazu Kagami, Hiroyuki Okamoto, Shuhei Sekii, Koji Inaba, Naoya Murakami, Hiroshi Igaki, Yoshinori Ito, Takashi Uno, Jun Itami

**Affiliations:** 1 Department of Radiation Oncology, National Cancer Center Hospital, 5-1-1 Tsukiji, Chuo-ku, Tokyo 104-0045 Japan; 2 Department of Radiation Oncology, Showa University Koto Toyosu Hospital, 5-1-38 Toyosu, Koto-ku, Tokyo 135-0061 Japan; 3 Department of Radiation Oncology, School of Medicine, Showa University, 1-5-8 Hatanodai, Shinagawa-ku, Tokyo 142-8666 Japan; 4 Department of Radiology, Chiba University, 1-33 Yayoi-cho, Inage-ku, Chiba-shi, Chiba 263-8522 Japan

**Keywords:** Breast cancer, Prone breast radiotherapy, Dose homogeneity, Acute radiation dermatitis

## Abstract

**Background:**

This prospective study aimed to compare dose volume histograms (DVH) of the breasts and organs at risk (OARs) of whole breast radiotherapy in the supine and prone positions, and frequency and severity of acute and late toxicities were analyzed.

**Methods:**

Early-stage breast cancer patients with large breasts (Japanese bra size C or larger, or the widest measurements of the bust ≥ 95 cm) undergoing partial mastectomy participated in this study. CT-based treatment plans were made in each position, and various dosimetric parameters for the breast and OARs were calculated to compare the supine and prone radiotherapy plans. The actual treatment was delivered in the position regarded as better.

**Results:**

From 2009 to 2010, 22 patients were prospectively accrued. Median follow-up period was 58 months. The homogeneity index and lung doses were significantly lower in the prone position (*P* = 0.008, *P* < 0.0001 and *P* < 0.0001, respectively). Cardiac dose showed no significant differences between two positions. By comparing two plans, the prone position was chosen in 77 % of the patients. In the prone position, ≥ grade 2 acute dermatitis were seen in 47 % of patients treated, whereas 20 % of the patients treated in the supine position had grade 2 and no cases of grade 3, although without a statistical significance of the rates of ≥ grade 2 acute dermatitis between the two positions (*P* = 0.28). The actual dose measurement using a breast phantom revealed significantly higher surface dose of the breast treated in the prone position than that in the supine position.

**Conclusions:**

Breast irradiation in the prone position improves PTV homogeneity and lowers doses to the OARs in the Japanese large-breast patients. However meticulous positioning of the breast in the prone board avoiding the bolus effect is necessary to prevent acute dermatitis.

## Background

Adjuvant whole breast radiotherapy (WBRT) after partial mastectomy for breast cancer is a gold standard. However, adjuvant WBRT may have technical difficulties in women with large breasts when treated in the supine position. Several institutions have shown increased radiation toxicities and worse cosmetic outcomes for patients with large, pendulous breasts and/or increased body mass index [1–4]. A previous study from our institution reported that the incidence of ≥ grade 2 acute dermatitis for the patients with large-volume breasts treated with WBRT were higher than for the other patients although without a statistical significance (15 % vs 7 %, *p* = 0.214) [5]. In addition, patients with large breasts may receive increased doses to critical structures such as the heart or the lungs owing to the breast positioning when the patients are treated in the supine position. WBRT in the prone position aims to overcome some of the technical limitations associated with treating large, pendulous breasts and/or large body habitus, and it may also reduce radiation doses to the organs at risk (OARs) [6–11].

Many reports of the prone WBRT have been published from the United States and Europe, but rarely from Japan. It is because the incidence of obesity in Japan is much lower than in the western countries and the number of Japanese patients with large breasts is small who would gain much benefit from WBRT in the prone position. However, in recent years, because of the changing dietary habits, breast size of Japanese women has become larger. In 1980 only 16.2 % of Japanese women had breasts of Japanese C cup brassiere or larger, in comparison to 62 % in 2004 [12], and the number of patients with large breasts is expected to increase further in the future, therefore an assessment of safety and efficacy of adjuvant prone WBRT in Japan deems to be necessary and important.

In this prospective study, we compared dose volume histograms (DVHs) of the breasts and OARs (heart and lung) in the supine and prone positions, and delivered actual treatment in the position which was regarded as better with respect to DVH. Furthermore, we investigated frequency and severity of acute radiation dermatitis and late toxicities in all the patients. Additionally, the difference in the surface doses between the two positions was analyzed by an actual dose measurement using a breast phantom.

## Methods

### Patient eligibility

The patients with stage 0-II (Tis-T2, N0-1) breast cancer and large or pendulous breasts (Japanese bra size C or larger, or the widest measurements of the bust equal to or over 95 cm) undergoing partial mastectomy in National Cancer Center Hospital were eligible for this prospective study. Exclusion criteria were history of irradiation to the ipsilateral breast, concurrent malignancy, and active connective tissue disorders. The patients with positive axillary nodes were required to undergo axillary lymph nodes dissection. Patients with four or more axillary lymph node metastasis were not eligible because the supraclavicular region was also irradiated routinely in ≥ 4 node-positive patients in our institution.

This prospective study was approved by the Institutional Review Board of the National Cancer Center (reference number: 21–15), and all enrolled patients gave their written informed consents before being registered in the study. Written informed consents for publication and presentation of individual clinical data had been obtained from all the participants.

### Simulation and target definition

**Fig. 1:**
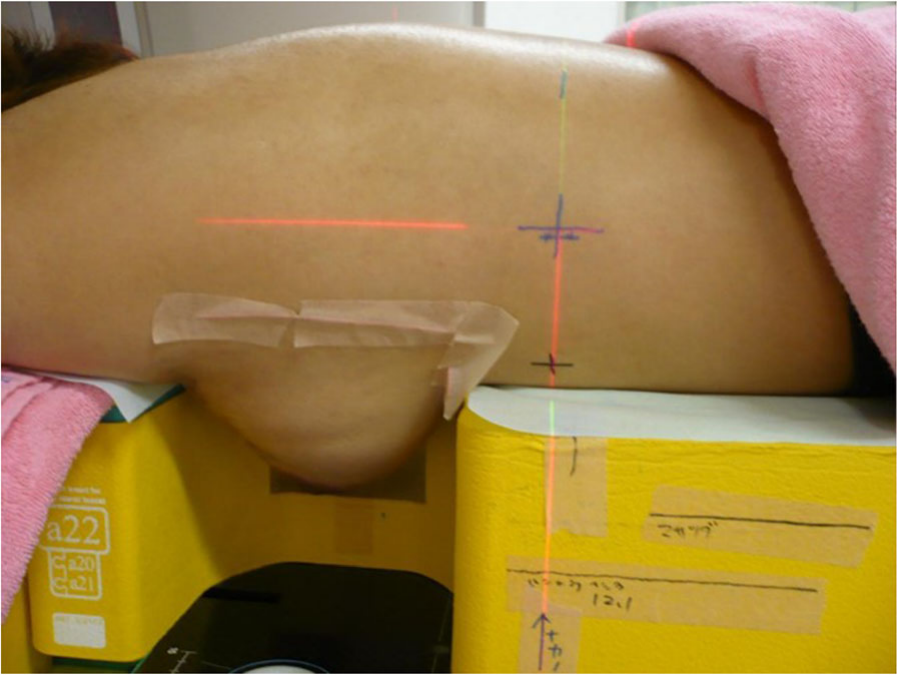
A patient lying on the prone board. Patients were simulated in the prone position in a prone board (ALL-IN-ONE patient positioning system, ORFIT, Wijnegem, Belgium) allowing the breast tissue to fall freely below the table

Each patient underwent two CT simulations (Aquilion™, Toshiba, Tokyo, Japan) in the supine and prone positions. Patients were first imaged (3-mm CT slice thickness) in the supine position with both arms over the head. The borders of the breast fields were marked using radioopaque wires. Patients were then reimaged in the prone position on a specially designed prone board (ALL-IN-ONE patient positioning system, ORFIT, Wijnegem, Belgium) that allowed the indexed breast tissue to fall freely below the table (Fig. 1).

Target and OARs (bilateral lungs and heart) were delineated on each CT slice in both positions. The clinical target volume (CTV) was defined as the entire ipsilateral palpable breast tissue, where the wires served as an aid to define the borders of the CTV. The CTV was assumed to start 5 mm below the skin. Postoperative cavity and all the clips placed during operation to show the cavity margins were included in the CTV. An isotropic 7 mm margin was added to the CTV to obtain the planning target volume (PTV). For evaluating the dose of the PTV, PTV_EVAL was generated from the PTV, excluding the lung and 2 mm thick tissue under the skin.

### Treatment planning

**Fig. 2:**
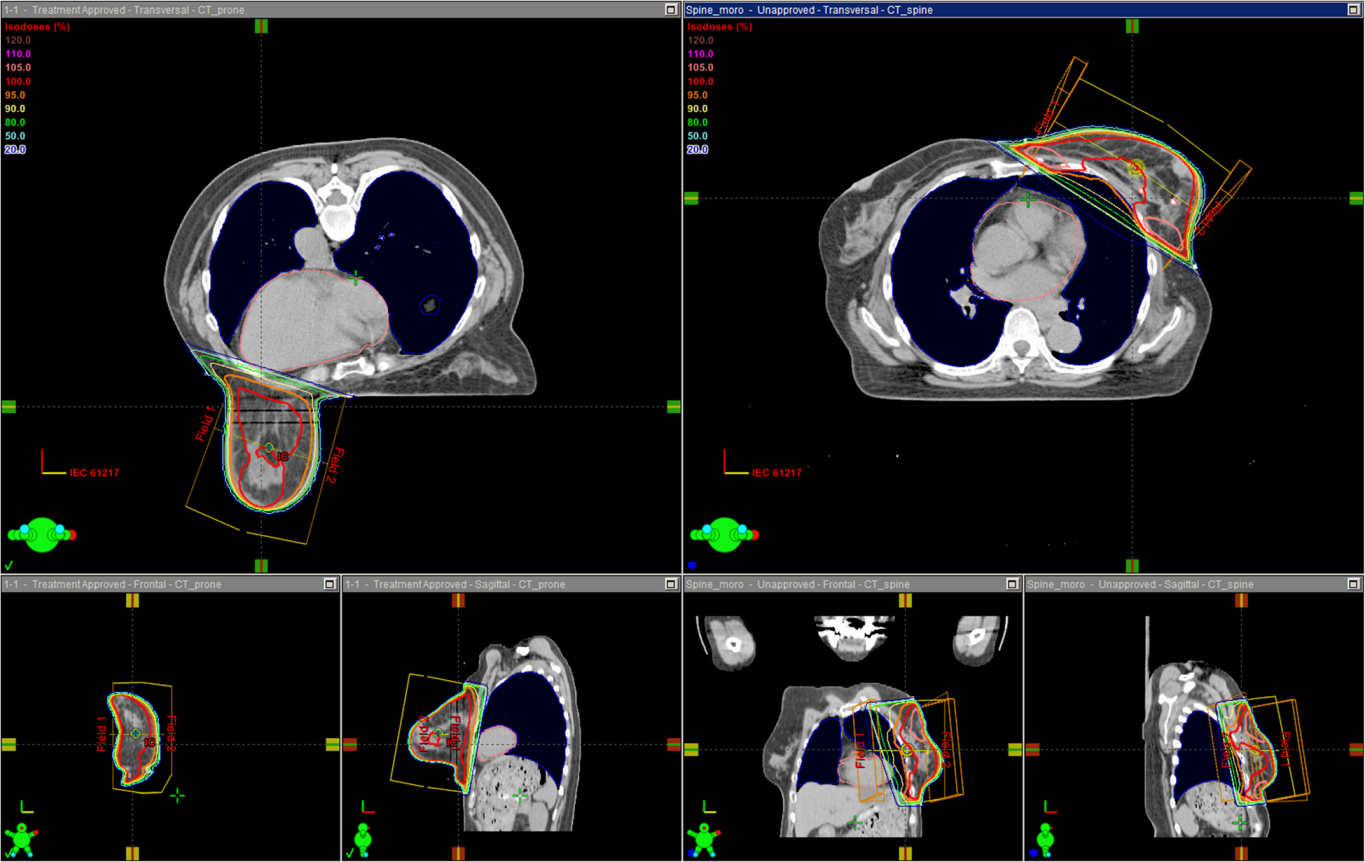
Typical dose distributions of a patients with a pendulous breast. For each patient, opposing tangential fields were setup to irradiate PTV in both supine and prone positions

For each patient, opposing tangential fields with 4 MV photons were setup to irradiate PTV in both supine and prone positions. Physical wedge filters were used when the maximum dose of PTV exceeded 115 % of the prescribed dose. A field-in-field technique was not allowed in this study because the breast shape in the prone position was not as reproducible as supine position. Beam edges of lung side were matched accordingly to reduce the lung dose. Radiation fields did not exceed the midline and did not include the contralateral breast. The dose prescribed to the ICRU prescription point was 50 Gy in 25 fractions. Beam data of 4 MV X-ray from a linear accelerator (Clinac iX, Varian, Palo Alto, CA, USA) was used for calculation of DVHs by Eclipse treatment planning system (Varian Medical Systems, Palo Alto, CA, USA). Figure 2 shows typical dose distributions of a patient with pendulous breasts in the prone and supine positions. Following dose parameters were calculated by using algorithm of the Analytical Anisotropic Algorithm (AAA) [13–15] with a heterogeneity correction: the minimum coverage dose of 5 % or 95 % of the PTV_EVAL (D_5%_, D_95%_), mean doses in the PTV_EVAL (D_mean_), bilateral lung volume irradiated equal to or over 20 Gy (lung V_20_), mean lung dose of the bilateral lungs (MLD), and mean heart dose. In an attempt to analyze dose homogeneity within the PTV_EVAL, homogeneity index (HI: D_5%_/D_95%_) was calculated for both positions.

In nine patients, boost irradiation of 10 Gy in five fractions by an electron beam was performed due to close margins, as defined at our institution as being < 5 mm. The electron irradiation was done in the supine position. In this study, the dose from electron irradiation was not taken into account.

### Selection of the better treatment position

DVHs of both positions were compared and the actual treatment was delivered in the position which was regarded as better with regard to DVH. The better treatment position could provide (1) better heart and lung sparing, and (2) improved dose homogeneity in PTV_EVAL, and it was determined by discussion of two radiation oncologists. In cases where the two radiation oncologists judged no DVH parameter differences in both positions, the supine position was chosen for the treatment because the supine position was more reproducible than the prone position.

### Study endpoints and statistical analysis

Primary endpoints of this study were the frequency and severity of acute radiation toxicities. In our institution, incidence of the acute morbidities including acute dermatitis ≥ grade 2 among the patients with large breast treated with supine WBRT was considered to be around 20 % [5]. Therefore, threshold incidence of the acute dermatitis ≥ grade 2 in the prone WBRT was assumed as 20 %, and the expected incidence as 7 %. With the type one error rate of 5 and 80 % power, 40 patients must be allocated to the prone WBRT, so the study will continue until 40 patients end up receiving prone WBRT.

Secondary endpoints were the comparison of PTV_EVAL dose homogeneity, doses to the OARs, and incidence of the late toxicities. The grade of acute dermatitis was classified according to the CTCAE, version 3 [16]. Acute dermatitis was graded by the worst toxicity occurring until 3 months after completion of the WBRT. Acute dermatitis was evaluated in the skin out of electron boost field. Late toxicity was assessed by LENT/SOMA [17]. Late toxicity was graded by worst toxicity from 4th month after WBRT to the last follow-up visit. Cosmetic outcome was physician-assessed at the last follow-up according to the Harvard Scale [18].

Statistical analyses were done using a two-sided paired *t*-test for continuous variables and chi-square test for categorical variables. For all statistical tests a significance level of 0.05 was used.

### Surface dose measurement of the breast phantom

In this study, incidence of ≥ grade 2 acute dermatitis in the prone WBRT was higher than estimated. Therefore, we performed an actual dose measurement using breast phantom in order to evaluate the skin dose. A single right breast phantom attached to the thorax phantom (Model 002LFC, CIRS, Virginia, USA) was used for the dose measurement. Thirty-five pieces of 2 cm × 2 cm cut-outs from a radiochromic film (EBT3, International Speciality Product, New Jersey, USA) were uniformly attached onto surface of the right breast phantom. After irradiation of 200 cGy using tangential fields with 4 MV X-rays, the 35 pieces of radiochromic film cut-outs were digitized with an ES-8500 flatbed scanner (SEIKO-EPSON, Nagano, Japan) under a resolution of 72 dpi. Absolute dose were derived from the optical density using a conversion table. Mean absolute dose was determined from measured values of four spots in the cut-out.

**Fig. 3:**
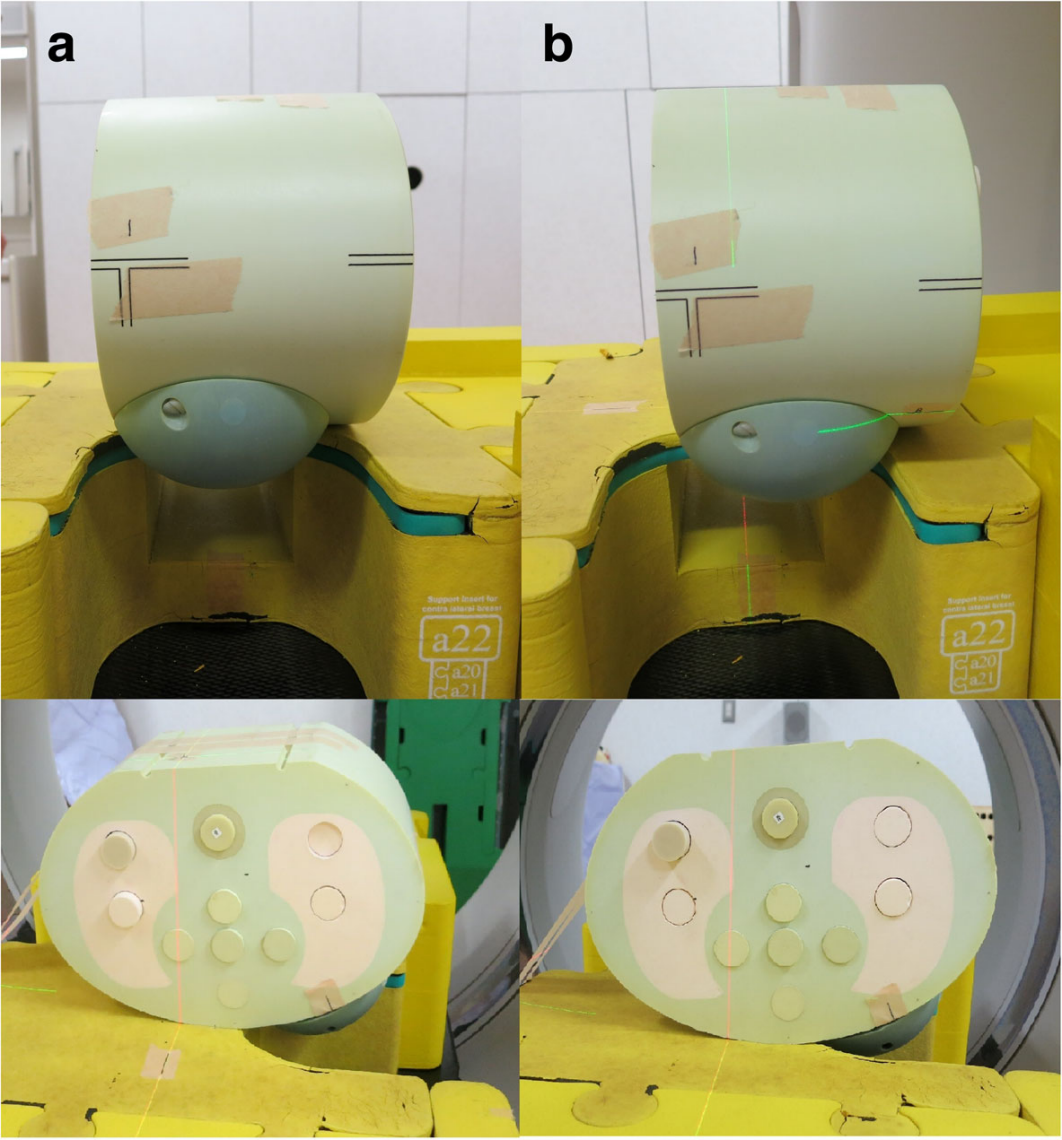
Two prone breast phantom positions. **a** The prone position where the breast phantom was located in the center of the prone board (“prone center position”). **b** The prone position where the breast phantom was located at the medial and cranial side of the prone board (“prone medial and cranial position”)

Dosimetry was performed in three breast phantom positions: the prone position where the breast phantom was located in the center of the prone board (“prone center position”), the prone position where the breast phantom was located at the medial and cranial side of the prone board (“prone medial and cranial position”) and the supine position (Fig. 3).

## Results

### Patient characteristics

Between September 2009 and May 2010, 22 patients with breast cancer undergoing partial mastectomy were prospectively accrued to this trial (13 right-sided: nine left-sided). Because of the unexpectedly high incidence of the acute dermatitis ≥ grade 2 in the prone WBRT, this trial was terminated after 17 patients underwent prone WBRT. Table 1 summarizes baseline characteristics of the 22 patients. Median age was 50 years (range: 35–74 years). More than half of the patients reported their bra cup-size as C. Five patients (23 %) with the tumor ≥ 3 cm or with positive lymph nodes received neoadjuvant chemotherapy. All three patients (14 %) with 1–3 lymph nodes metastases underwent axillary lymph nodes dissection and received adjuvant or neoadjuvant chemotherapy. No patients had four or more lymph nodes metastases, therefore radiation fields including the axillary or the supraclavicular region were not used in this study. All patients completed the prescribed course of external beam radiotherapy. None of the patients required a treatment break due to acute toxicity. Median follow-up length was 58 months (range: 20 to 64 months).

**Table 1:** Characteristics of 22 patients in the study

Breast	Right	13 (59 %)
	Left	9 (41 %)
Age (years)	Median (Range)	50 (35–74)
ECOG PS 0		22 (100 %)
Self-reported Japanese bra cup-size	B	2 (9 %)
	C	12 (55 %)
	D	4 (18 %)
	E	2 (9 %)
	F	2 (9 %)
Stage	0	4 (18 %)
	IA	7 (32 %)
	IIA (T2N0)	8 (36 %)
	IIB (T2N1)	2 (9 %)
	IIIA (T3N1)	1 (5 %)
Tumor size (cm)	Median (Range)	2.2 (0.6–5.1)
Neoadjuvant chemotherapy	Yes	5 (23 %)
	No	17 (77 %)
WBRT dose (50 Gy)		22 (100 %)
Boost radiation (10Gy)	Yes	9 (41 %)
	prone (*n* = 17)	6 (35 %)
	supine (*n* = 5)	3 (60 %)
	No	13 (59 %)
Follow-up (months)	Median (Range)	58 (20–64)

*ECOG* Eastern Cooperative Oncology Group, *PS* performance status, *WBRT* whole breast radiotherapy

### Treatment-related toxicities and cosmetic results

The prone position was chosen in 17 (77 %) patients and the supine position was chosen in 5 (23 %) patients for WBRT as described below in detail. Acute dermatitis of the patients treated in the prone position was grade 1 in 9/17 (53 %), grade 2 in 7/17 (41 %), and grade 3 in 1/17 (6 %). For patients treated in the supine position, there were no cases of grade 3 dermatitis, while 4/5 (80 %) had grade 1, 1/5 (20 %) had grade 2 (Table 2). There were no cases of acute dermatitis ≥ grade 4 in both treatment groups. Incidence of ≥ grade 2 acute dermatitis was higher in the prone position although without a statistical significance (*P* = 0.28). The most frequent late toxicity was pigmentation, which occurred in 35 % of patients treated in the prone position and 20 % in the supine position. Severity of the late toxicities were limited to grade 1 or 2 in all the patients. No patients experienced breast fibrosis or breast retraction. There were no cases of symptomatic radiation pneumonitis or significant cardiac events during the follow-up period in both treatment groups.

**Table 2:** Acute dermatitis, late toxicity and physician-assessed cosmesis in the prone and supine positions

Toxicity	Prone (*n* = 17)	Supine (*n* = 5)
Acute dermatitis		
Grade1	9 (53 %)	4 (80 %)
Grade2	7 (41 %)	1 (20 %)
Grade3	1 (6 %)	0
Late toxicity		
Pigmentation		
Grade1	5 (29 %)	1 (20 %)
Grade2	1 (6 %)	0
Fibrosis		
Grade1	0	0
Grade2	0	0
Retraction		
Grade1	0	0
Grade2	0	0
Telangiectasia		
Grade1	1 (6 %)	0
Grade2	0	0
Edema		
Grade1	2 (12 %)	1 (20 %)
Grade2	0	0
Cosmesis		
Execellent/Good	16 (94 %)	5 (100 %)
Fair	1 (6 %)	0
Poor	0	0

On the basis of the Harvard Scale for cosmetic outcomes, the majority of patients (94 % in the prone position, and 100 % in the supine position) had good or excellent cosmetic outcomes (Table 2). Only one patient treated in the prone position had a fair cosmetic outcome (6 %).

### DVH analysis

CT-based treatment plans were made in two treatment positions (supine/prone) and the DVHs of PTV_EVAL and OARs (lung, heart) were compared. In the prone position, D_5%_ was significantly lower (*P* = 0.004) and D_95%_ was significantly higher (*P* = 0.01) than in the supine position, but D_mean_ and the volume of PTV_EVAL were not different between the two positions (D_mean_: *P* = 0.53, the volume of PTV_EVAL: *P* = 0.74). The homogeneity index was significantly lower for the prone position (mean 1.16) than for the supine (mean 1.27) (*P* = 0.008) (Table 3).

**Table 3:** Volumes and dosimetric values of PTV_EVAL and OARs in the prone and supine positions

	Prone	Supine	*p*
	Mean ± SD		
PTV_EVAL			
PTV_EVAL volume (cm^3^)	629 ± 252	636 ± 247	0.74
D_5%_ (Gy)	52.3 ± 0.8	53 ± 1	0.004
D_95%_ (Gy)	45.2 ± 1.4	42.3 ± 4.8	0.01
Dmean (Gy)	48.9 ± 1.8	48.6 ± 1.5	0.53
HI	1.16 ± 0.04	1.27 ± 0.19	0.008
OARs			
Lung V_20_ (%)	0.8 ± 0.8	4.6 ± 1.7	<0.0001
Mean lung dose (Gy)	1.4 ± 0.6	3.6 ± 0.8	<0.0001
Mean heart dose (Gy) (*n* = 9)	3.1 ± 1.6	3.0 ± 0.9	0.9

*HI* homogeneity index (D_5%_/D_95%_), *OARs* organs at risk, *PTV_EVAL* planning target volume for evaluation

The prone position afforded a greater sparing of the lung. Mean lung V_20_ and MLD were lower in the prone position with a statistical significance (lung V_20_: *P* < 0.0001, MLD: *P* < 0.0001) (Table 3). Cardiac dose was evaluated in the nine patients with left-sided cancers; there were no significant differences between the two positions (*P* = 0.9) (Table 3).

### Treatment position

By comparing two treatment plans, the prone position was chosen in 17 (77 %) patients, because it spared the lung better in 17/17 (100 %), homogeneity or coverage of PTV_EVAL were better in 7/17 (41 %), or heart dose was lower in 2/17 (12 %). In the remaining 5 (23 %) patients, the supine position was chosen for the treatment, because it enabled better heart exclusion from the fields in 1/5 (20 %) and PTV_EVAL homogeneity was better in 1/5 (20 %). In the patients with no differences of dose parameters in both positions (3/5; 60 %), the supine position was chosen for the treatment.

### Treatment efficacy

During the follow-up, no locoregional recurrence occurred among the 22 patients. Three patients developed distant failures; one of these patients expired, and two are currently alive with disease.

### Surface dose measurement of the breast phantom

**Fig. 4:**
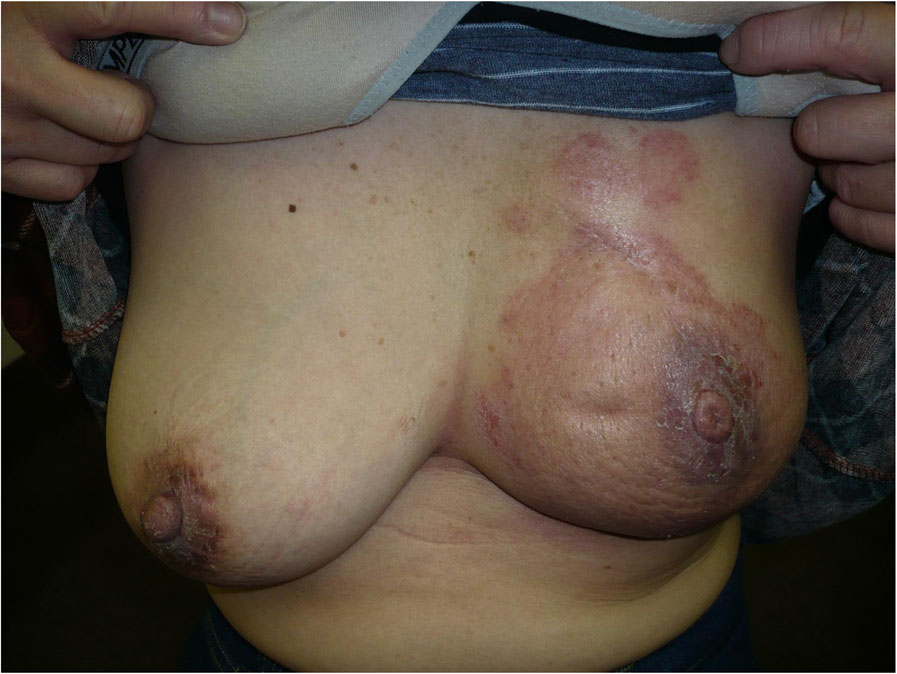
Acute dermatitis in the medial part of the irradiated breast. We found unexpectedly that the patients treated in the prone position had a higher tendency to develop acute dermatitis in the medial part of the ipsilateral breast

We found unexpectedly that the patients treated in the prone position had a higher tendency to develop acute dermatitis in the medial part of the ipsilateral breast (Fig. 4). We hypothesized that the unusual distribution of acute dermatitis could be explained by a bolus effect of the prone board. To validate the hypothesis, we performed an actual dose measurement using a breast phantom and the prone board.

Dose measurement was performed in the three breast phantom positions as described above. Because the breast phantom was not large or pendulous, the “prone medial and cranial position” was supposed to reproduce the situation that the large or pendulous breast was pressed into the edge of the prone board.

**Fig. 5:**
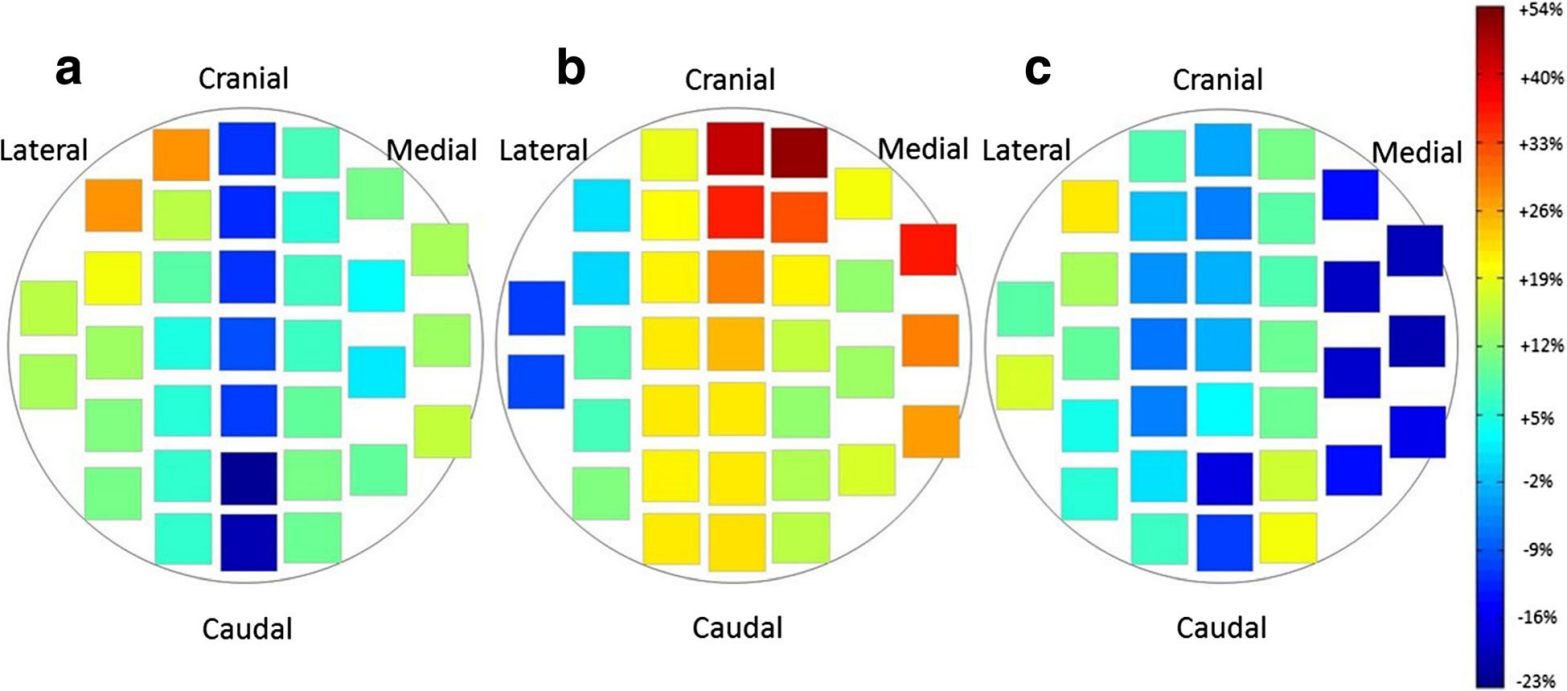
Color map showing the dose of each film cut-out piece. **a** “Prone center position”. **b** “Prone medial and cranial position”. **c** Supine position. Medial breast surface in the “prone medial and cranial positreferenion” was irradiated to the highest dose

The surface dose of the breast phantom was significantly higher in both prone positions than in the supine position. (“prone center position” vs supine position: *P* = 0.01, “prone medial and cranial position” vs supine position: *P* < 0.0001) (Table 4). Furthermore, surface dose of the breast phantom was significantly higher in the “prone medial and cranial position” than that in the “prone center position” (*P* = 0.0007). Figure 5 shows a higher surface dose in medial and cranial part of the right breast phantom treated in the “prone medial and cranial position”. This part corresponds approximately to the pressed breast area to the prone board and this dose distribution might be consistent with the unusual distribution of acute dermatitis in the prone WBRT.

**Table 4:**
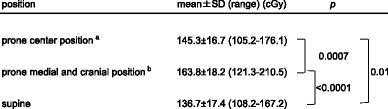
Surface doses of the breast phantom

^a^prone center position: Prone position where the breast phantom was located in the center of the prone board

^b^prone medial and cranial position: Prone position where the breast phantom was located at the medial and cranial side of the prone board

## Discussion

Dose inhomogeneity of tangential WBRT has been implicated in the occurrence of poor cosmetic outcomes and late toxicities in the patients undergoing partial mastectomy and postoperative WBRT. Several authors have published data demonstrating that the prone position for WBRT improves dose homogeneity of the irradiated breast and limits the dose to the OARs (Table 5). However, this study is the first prospective trial delivering actual postoperative radiotherapy in the position which was regarded as better than the other after comparing dose parameters both in the prone and supine positions.

**Table 5:** Comparison of published series of prone position for breast radiotherapy

References	Year	Number	Study objectives	Inclusion criteria	Observated results
Merchant et al. [10]	1994	56	Prone whole breast iradiation	Breast irradiation	Improve dose homogeneity of the breast
Grann et al. [19]	2000	56	Prone whole breast iradiation	Large or pendulous breast	Improve dose homogeneity of the breast. Eighty percent of patients experienced Grade I or Grade II erythema.
Mahe et al. [20]	2002	35	Prone whole breast iradiation	Large and/or pendulous breast	The high-dose regions of the base and the top of the breast did not exceed 105 %. Only G1-2 acute dermatitis was observed.
Griem et al. [8]	2003	15	Planning comparison prone vs. supine	Breast irradiation	Improve dose homogeneity with the prone position. Significant improve lung DVH, no differences for heart.
Formenti et al. [7]	2004	50	Partial breast irradiation in prone	Postmenopausal T1N0	Good lung and heart DVH
Buijsen et al. [6]	2007	10	Planning comparison prone vs. supine	Pendulous breasts (bra sizeD and over)	Improve dose homogeneity and lung DVH with the prone position
Stegman et al. [29]	2007	245	Prone whole breast iradiation	Beams with gantry angles of 90° ± 10°and 270° ± 10°	Grade 2–3 acute dermatitis were limited to 18 %. Grade 2, Grade 3, and Grade 4 chronic dermatitis was seen in 27.8, 2.8, and 1.6 %.
Varga et al. [11]	2009	61	Planning comparison prone vs. supine	Breast irradiation	Significant improve lung DVH, no differences for heart.
Kirby et al. [24]	2010	65	Planning comparison prone vs. supine	Partial or total breast irradiation	Improve lung DVH; improve heart DVH for big breast
Bergom et al. [21]	2012	110	Prone whole breast iradiation	Large body habitus and/or large-pendulous breasts	Excellent to good cosmesis was achieved in 89 %. G3 acute dermatitis in 5 %.
Lymberis et al. [25]	2012	100	Planning comparison prone vs. supine (3DCRT or IMRT)	Breast irradiation	Improve lung and heart DVH with the prone position
Formenti et al. [26]	2012	200	Planning comparison prone vs. supine	Breast irradiation	Reduction in the amount of irradiated lung in all patients and in the amount of heart volume irradiated in 85 % of patients with left breast cancer.
Mulliez et al. [22]	2013	100	Comparing prone and supine setup of hypo-fractionated IMRT	European cup size C or more	Improve dose coverage, better homogeneity, less volumes of over-dosage with the prone position

*DVH* dose volume histograms, *3DCRT* Three-dimensional conformal radiation therapy, *IMRT* Intensity Modulated Radiation Therapy

In this study, we could demonstrate improvement of the dose homogeneity in the prone position for large breasted patients. Reports from larger series have indicated the same trend as this study [8, 10]. The benefit was more evident in patients with very large or pendulous breasts, or deformities of the chest cavity [6, 19, 20]. Our criteria of large breast was different from the ones previously reported from the western countries. Japanese bra size is different from the US size. For example, most Japanese C cup corresponds to US B cup. The breast volumes of our patients were smaller than that of the patients enrolled in the western studies where mean breast volumes were more than 1000 cm^3^ [21, 22], while the mean breast volume of our patients was 629 cm^3^ (range 230–1074 cm^3^). Even if the breast size was not as large as the ample bust of women in the US or Europe, improvement of the dose homogeneity was demonstrated in the PTV_EVAL by using prone position.

One of the serious problems in treatment of the large breasts is the larger lung volumes irradiated due to the steep gantry angles needed to obtain an adequate coverage of breast tissue. Even in the smaller breasts in our study, lung V_20_ and MLD, an indicator for radiation-induced lung damage, showed significantly lower values in the prone position as compared to the supine position. This was in accordance with other studies that analyzed lung dose in the prone breast irradiation [6, 7].

As shown by the large study of Darby et al. [23], larger incidental dose to the heart increased the risk of ischemic heart disease especially in women with preexisting cardiac risk factors. Although we could evaluate cardiac dose of only nine patients with left-sided cancers, there were no significant differences between the two positions. The dose to the heart is generally not higher in the prone as compared to the supine WBRT [8, 11, 24, 25]. Formenti et al. [26] reported that the prone position was associated with a reduction of in-field heart volume compared with the supine position, but the reduction reached a statistical significance only in the women with breast size ≥ 750 cm^3^. Probably because of the small number and the small breast volume of our patients, difference of the cardiac doses might not be statistically significant between the two positions. However, the dose to the coronary arteries, left ventricle or the anterior compartment might increase due to displacement of the heart anteriorly in the prone position [27]. Optimal sparing of coronary arteries by contouring of left anterior descending branch is recommended if patients are treated with the prone WBRT [28].

Despite improved dose homogeneity and DVHs of OARs in the prone position, we found the patients actually treated in the prone position developed severer dermatitis than we had expected. In our study, 47 % of patients treated in the prone position experienced grade 2–3 acute dermatitis. In contrary, results of the previous studies have indicated that the proportion of patients with severe acute dermatitis was small even when treated in the prone position [19–21, 29]. By measuring actual dose using a breast phantom, we could indicate the surface dose of the breast where it is pressed to the prone board was higher than other areas of the breast. It was due to the bolus effect of prone board, because high dose to the cranial medial side of the phantom was not seen in the irradiation in the “prone center position”, where surface of the breast phantom was not pressed to the prone board. We also demonstrated a slightly higher surface dose of the breast treated in the “prone center position” compared to the supine position with a statistical significant difference. The “prone center position” is corresponding to the setup using prone board. Because the difference of the setups between the “prone center position” and supine position was presence or absence of the prone board, we assumed that the bolus effect of the prone board occurred even though it was not abutted the breasts in the “prone center position”. We routinely use 4 MV photons when setting up tangential radiation fields of the breast because the small breasts are common in Japan, and accordingly we used 4 MV photons in this study. Meanwhile, more than 6 MV photons are usually used for setup to irradiate large breasts in US and Europe. The bolus effect is typically more apparent when using 4 MV photons than using over 6 MV photons, therefore in this study, higher surface dose of breast phantom was clearly demonstrated and severer dermatitis with an unusual distribution was observed, which differed from the toxicity results of the previous studies. We recommend that breast surface, especially of the cranial and medial side, should not be attached and pressed to the prone board in case of the prone WBRT and 6 MV or higher energy photon should be used to treat patients with large or pendulous breast.

**Fig. 6:**
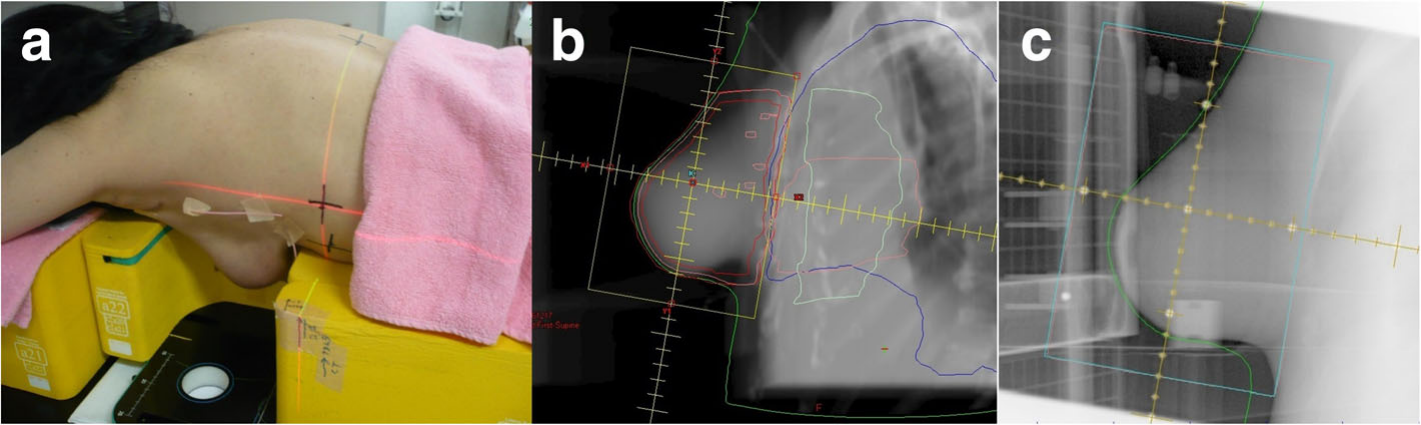
Prone setup of the patient shown in the Fig. 4. **a** The setup image. **b** Digitally reconstructed radiography (DRR). **c** Verification portography

Figure 6 shows the setup image, digitally reconstructed radiography (DRR) and verification portography of the patient shown in the Fig. 4, and the setup position of this patient seemed to be acceptable. In our institution, the verification portography was taken only once on the first day of WBRT due to an abundance of breast cancer patients. The prone position is not as reproducible as the supine position and sometimes difficult to set up even using markers of body surface. In order to reduce interfractional positioning difference, verification portography should be monitored more frequently when the patient is treated in the prone position than in the supine position.

Incidence of non-serious late toxicities and cosmetic results were not different between the prone and supine positions.

The major limitation of this study is that the number of enrolled patients was small; this was because the population of patients with large breasts was unexpectedly small, and we experienced relatively unexpected acute dermatitis among the patients treated in the prone position during registration. We performed axillary lymph node dissection on all node-positive patients and they didn’t receive regional nodal irradiation. Since MA.20 [30] and EORTC 22922/10925 [31] were reported, the trend in treatment for 1–3 node-positive patients have been an addition of irradiation to the supraclavicular nodes and parasternal nodes. Additionally, the results from ACOSOG Z0011 [32] and AMAROS [33] indicated the omission of axillary lymph nodes dissection had a low impact on the local recurrence or prognosis when appropriate cases were selected and adequate adjuvant therapy including regional nodal irradiation were performed. The present study was conducted before the results of studies described above were available, thus the patients who might have to receive regional nodal irradiation were treated without regional irradiation. However, all the patients with positive nodes in our study underwent axillary lymph nodes dissection and adjuvant or neoadjuvant chemotherapy, and no recurrence of regional lymph nodes were observed during follow-up.

The strengths of our study include that toxicity and cosmesis were scored prospectively, and the median follow-up length of 58 months was sufficiently long to take into account the latency of radiation toxicities. The breast surface dose was measured using breast phantom resulting in the confirmation that the surface dose was higher treated in the prone position than treated in the supine position. With a meticulous positioning of the breast in the prone board, an appropriate choice of photon energy according to the size of the breast, and verification portography with a reasonable frequency, acute dermatitis can be prevented and prone WBRT will be a preferred technique to improve PTV_EVAL homogeneity and OAR doses in a large breast.

## Conclusions

WBRT for Japanese large-breasted women in the prone position will improve PTV_EVAL homogeneity and OAR doses. However if a prone board is used, a meticulous positioning of the breast, an appropriate choice of photon energy and verification portography of setup with a reasonable frequency is necessary to prevent severe acute dermatitis.

## Abbreviations

AAA: Analytical Anisotropic Algorithm
CTV: Clinical target volume
D_mean_: Mean doses in the PTV_EVAL
D_5%_: Minimum coverage dose of 5 % of PTV_EVAL
D_95%_: Minimum coverage dose of 95 % of PTV_EVAL
DVH: Dose volume histograms
HI: Homogeneity index
lung V_20_: Bilateral lung volume irradiated equal to or over 20 Gy
MLD: Mean lung dose of bilateral lungs
OAR: Organ at risk
PTV: Planning target volume
PTV_EVAL: Planning target volume for evaluation
WBRT: Whole breast radiotherapy
